# Investigation of female survival benefit in metastatic melanoma

**DOI:** 10.1038/sj.bjc.6690637

**Published:** 1999-08

**Authors:** B Richardson, A Price, M Wagner, V Williams, P Lorigan, S Browne, J G Miller, S Mac Neil

**Affiliations:** University Department of Medicine and Departments of Clinical Chemistry and Plastic and Reconstructive Surgery, Northern General Hospital Trust, Sheffield, S5 7AU, UK; Department of Clinical Oncology, Weston Park Hospital, Sheffield, S10, UK

**Keywords:** melanoma, invasion, oestrogen, testosterone, oestrogen receptor-β

## Abstract

Epidemiological studies show female survival benefit in advanced metastatic melanoma. In investigating a possible mechanism for this female survival benefit, we have previously reported that the female steroid 17β-oestradiol significantly reduces invasion of a human melanoma cell line (A375-SM cells) and ocular melanoma cells through fibronectin. Neither cell type was found to possess oestrogen receptor-α. The aim of the current study was to obtain further information on the extent to which progression of cutaneous melanoma might be sex steroid sensitive by (a) examining the relationship between circulating sex steroids, sex hormone binding globulin and disease progression; (b) examining the relationship between sex steroid structure and the ability of steroids to reduce invasion of a melanoma cell line in vitro; and (c) examining the effects of sex steroids on proliferation of these cells in vitro. We report a significant reduction in circulating oestrone with disease progression in male but not female patients. Examining steroids for their ability to inhibit invasion of A375-SM cells through fibronectin in vitro, oestrogenic compounds (17β-oestradiol and oestrone) were found to inhibit invasion; in this respect, oestrone was approximately 50 times more potent than 17β-oestradiol; steroids lacking the benzene ring structure did not inhibit invasion, indeed dehydroepiandrosterone (DHEA) which acts as a precursor to androgenic steroids significantly enhanced invasion. Proliferation of A375-SM cells was unaffected by 17β-oestradiol, oestrone or dihydrotestosterone when cells were cultured on plastic; in contrast, all three steroids induced modest proliferation of cells when grown on fibronectin with dihydrotestosterone the most mitogenic of the three steroids. These data are consistent with sex steroids playing a role in melanoma progression. © 1999 Cancer Research Campaign


*British Journal of Cancer* (1999) **80**(12), 2025–2033
© 1999 Cancer Research Campaign
Article no. bjoc.1999.0637

### Investigation of female survival benefit in metastatic melanoma

**B Richardson[1], A Price[2], M Wagner[1], V Williams[1], P Lorigan[4], S Browne[4], JG Miller[3] and S Mac Neil[1]**

[1]University Department of Medicine and Departments of [2]Clinical Chemistry and [3]Plastic and Reconstructive Surgery, Northern General Hospital Trust, Sheffield S5 7AU, UK; [4]Department of Clinical Oncology, Weston Park Hospital, Sheffield S10, UK

**Summary** Epidemiological studies show female survival benefit in advanced metastatic melanoma. In investigating a possible mechanism for this female survival benefit, we have previously reported that the female steroid 17β-oestradiol significantly reduces invasion of a human melanoma cell line (A375-SM cells) and ocular melanoma cells through fibronectin. Neither cell type was found to possess oestrogen receptor-α. The aim of the current study was to obtain further information on the extent to which progression of cutaneous melanoma might be sex steroid sensitive by (a) examining the relationship between circulating sex steroids, sex hormone binding globulin and disease progression; (b) examining the relationship between sex steroid structure and the ability of steroids to reduce invasion of a melanoma cell line in vitro; and (c) examining the effects of sex steroids on proliferation of these cells in vitro. We report a significant reduction in circulating oestrone with disease progression in male but not female patients. Examining steroids for their ability to inhibit invasion of A375-SM cells through fibronectin in vitro, oestrogenic compounds (17β-oestradiol and oestrone) were found to inhibit invasion; in this respect, oestrone was approximately 50 times more potent than 17β-oestradiol; steroids lacking the benzene ring structure did not inhibit invasion, indeed dehydroepiandrosterone (DHEA) which acts as a precursor to androgenic steroids significantly enhanced invasion. Proliferation of A375-SM cells was unaffected by 17β-oestradiol, oestrone or dihydrotestosterone when cells were cultured on plastic; in contrast, all three steroids induced modest proliferation of cells when grown on fibronectin with dihydrotestosterone the most mitogenic of the three steroids. These data are consistent with sex steroids playing a role in melanoma progression.

**Keywords**: melanoma; invasion; oestrogen; testosterone; oestrogen receptor-β

Although the acquisition of melanoma does not seem to be influenced by female sex hormones (Gefeller et al, 1998), the outcome of established melanoma does appear to be influenced by endocrine status. Female patients with malignant melanoma have some survival advantage as has become evident from recent multivariate analysis of large databases (reviewed in Miller and Mac Neil, 1997). Some investigators have suggested a survival advantage in pre- as opposed to post-menopausal women (Shaw et al, 1980; Karjalainen et al, 1988; Cocconi et al, 1992) but in another large multi-variate analysis (Stidham et al, 1994) the female advantage was equally strong in pre- and post-menopausal groups.

If female steroids confer some advantage in established melanoma then one might expect this to be most evident in pregnancy. However, the literature concerning melanoma progression in pregnancy presents a complex picture (MacKie et al, 1991). Pregnancy is often associated with hyperpigmentation of the skin, clearly indicating an influence of steroids on melanocyte biology. There are reports of melanoma progression being exacerbated by pregnancy (Slinguff and Seigler, 1992); paradoxically there are also reports of reduced risk associated with early childbearing and multi-parity (Shaw et al, 1978). One point on which studies of melanoma in pregnancy do agree is that pregnancy-associated melanomas are often significantly thicker than that of non-pregnancy-associated tumours. Intriguingly these thicker tumours are not necessarily associated with a less favourable prognosis than tumours arising in a non-pregnant woman (Travers et al, 1995).

In animal studies, there are a small number of reports indicating a female survival benefit with metastatic melanoma. In one study using severe combined immunodeficient (SCID) mice, melanoma cells produced liver metastases preferentially in male compared with female mice (Ladanyi et al, 1995). A study using athymic mice showed that oestrogen receptor-positive human melanomas grew more slowly in females than in male mice (Feucht et al, 1988) and, intriguingly, dihydrotestosterone has been reported to stimulate melanoma cell proliferation (Morvillo et al, 1995). In the latter study increased survival of male nude mice transplanted with melanoma tumours was seen when mice were given the anti-androgen hydroxyflutamide.

The mechanism of the apparent female survival benefit in melanoma has not been investigated in any great depth as yet nor have its implications been exploited with respect to prevention of initial metastasis. The depth of extension of malignant melanocytes into the dermis is a key factor in prognosis of malignant melanoma (Breslow et al, 1970). At present, patients presenting with thin tumours undergo surgical excision but are not usually offered any prophylactic treatment. With this objective in mind, we recently examined patients' sex steroid status and disease progression and identified a significant decrease in sex hormone binding globulin with disease progress in male but not female patients (Miller et al, 1997). However, this particular study had relatively few patients with advanced metastatic disease. In vitro we found clear anti-invasive effects of 17β-oestradiol when we examined invasion of a cutaneous melanoma cell line through fibronectin (Dewhurst et al, 1997), despite the fact that the

*Received 8 September 1998*
*Revised 19 February 1999*
*Accepted 23 February 1999*

*Correspondence to:* S Mac Neil

A375-SM cell line used in this study did not possess classical oestrogen receptors (oestrogen receptor-α) nor did the majority of melanoma tumours which we investigated (Dewhurst et al, 1997, and Miller et al, 1997, respectively).

In the current study we have continued investigations of sex steroid status and tumour progression for a cohort of patients with advanced metastatic disease versus appropriately aged and sex-matched controls and we have investigated to what extent the anti-invasive effect of 17β-oestradiol is specific to oestrogenic steroids.

We report a modest reduction in sex hormone binding globulin and oestrone with disease progression in male but not female patients. Examining a range of steroids for anti-invasive activity, we found oestrogenic compounds (17β-oestradiol and oestrone) to inhibit invasion; steroids lacking the benzene ring structure did not inhibit invasion, indeed dehydroepiandrosterone (DHEA), which acts as a precursor to androgenic steroids, significantly enhanced invasion of A375-SM cells through fibronectin by 40%. Dihydrotestosterone also significantly stimulated proliferation of these cells when grown on fibronectin but not when grown on tissue culture plastic.

### MATERIALS AND METHODS

Fibronectin (from human plasma), trypsin–EDTA, newborn calf serum, basal medium eagle (BME-phenol free), insulin, transferrin, 17β-oestradiol (water soluble), oestrone, cortisol, sodium bicarbonate, DHEA, progesterone, androstenedione, testosterone and MTT (3-[4,5-dimethylthiazol-2-yl]-2,5-diphenyltetrazolium bromide) were obtained from Sigma Chemical Company (Poole, Dorset, UK). Penicillin–streptomycin, L-glutamine, vitamin concentrate, non-essential amino acids, amphoteracin B (fungizone), sodium pyruvate, Dulbecco's modified Eagle's medium (DMEM) (with phenol red indicator), RPMI-1640 (both with and without phenol red indicator), DMEM: nutrient mixture F-12 Ham (1:1 mixture), nutrient mixture F-10 Ham were obtained from Gibco BRL (Paisley, UK). Essential Modified Eaglés Medium (EMEM) (10 × concentrate) was obtained from ICN Pharmaceuticals (Oxfordshire, UK). Fetal calf serum (FCS) was obtained from APP (West Midlands, UK). Transwell inserts were obtained from Costar UK (High Wycombe, Buckinghamshire, UK).

### Cell lines and culture conditions

The human cutaneous melanoma cell line A375-SM was a generous gift from IJ Fidler (USA) via MJ Humphries (University of Manchester, UK). These cells were maintained by serial passages in EMEM supplemented with pencillin (100 U $ml^{-1}$), streptomycin (100 μg $ml^{-1}$), Fungizone (1.2 μg $ml^{-1}$), L-glutamine (2 μM), sodium pyruvate (1 mM), vitamin concentrate (1.5% of 100 × stock), non-essential amino acids (1%), sodium bicarbonate (0.187%) and FCS (10%) at 37°C in a 5% carbon dioxide 95% air atmosphere. Their doubling time was 20 h.

The human cutaneous melanoma cell line HBL was a generous gift of Professor G Ghanem (Laboratory of Oncology & Experimental Surgery, Université Libre de Bruxelles, B-1000 Brussels). These cells were maintained by serial passage in nutrient mixture F-10 Ham supplemented with penicillin (100 U $ml^{-1}$), streptomycin (100 μg $ml^{-1}$), L-glutamine (2 μM), FCS (5%) and newborn calf serum (5%). Their doubling time was around 24 h.

### Invasion assay

The invasion assay used was as recently described from this laboratory (Dewhurst et al, 1997). Transwell inserts, containing a polycarbonate filter with 8-μm-diameter pores randomly distributed over its surface, were inverted and 50 μl of human fibronectin (at 100 μg $ml^{-1}$) added to underside of the polycarbonate filter and left for 1 h at 37°C in a 5% carbon dioxide 95% air atmosphere. The inserts were then placed the correct way up in wells of a 24-well plate containing 600 μl of the serum-free medium. All cell types used in the study were removed from the tissue culture flasks using 0.5 g $l^{-1}$ trypsin–0.2 g $l^{-1}$ EDTA, centrifuged at 250 ***g*** for 5 min and then resuspended in serum-free medium.

Cell suspensions (50 μl containing approximately $1.2 \times 10^5$ cells) plus an equivalent volume of serum-free medium (with or without drug) were then added to each transwell. (In studies examining the effect of different media on cell invasion, cells were cultured in the medium in question for 2 days prior to investigation of the effects of this medium (minus FCS) on steroid inhibition of invasion). Cells were then left for 20 h at 37°C in a 5% carbon dioxide 95% air atmosphere. The time of 20 h was chosen as, by this time, sufficient invasion had occurred, and it was below the doubling time of the cells used. Following examination under the light microscope, all cells in the experiment were counted using the following techniques. The medium containing cells was collected from the upper and lower chambers and replaced with an equivalent volume of trypsin–EDTA to remove the remaining cells from the assay. Cell number (of trypsinized cells plus any in the media in the upper and lower chambers) was then determined using a haemocytometer. Invaded cells were counted as those removed from the underside of the filter, suspended in the medium of the 24-well plate or attached to the bottom of the well. Non-invaded cells were counted as those remaining in the transwell or attached to the upper surface of the filter. The percentage of the total amount of cells that had invaded through fibronectin over 20 h was then calculated.

### Cell proliferation

The MTT-eluted stain assay (MTT-ESTA) was used to examine changes in cell number as previously described from this laboratory (Mac Neil et al, 1993). This colourimetric assay is based on assessing cellular dehydrogenase activity which indirectly reflects cell number. In these experiments, cells were plated on either tissue culture plastic or fibronectin in the presence and absence of 10% FCS.

### Dilution of steroids

Dilutions of aliquoted steroids were made up as 10 mM stocks in ethanol and stored at 4°C until required for use. Steroids were then diluted in serum-free media as required. All steroids were examined at concentrations which spanned their physiological range and did not exceed it by more than 1 log order.

### Selection of patients and control subjects

Patients who were approached to join the study were those attending the Weston Park Hospital Out-Patients Clinic. All volunteer patients gave written consent for the study under a protocol

 © 1999 Cancer Research Campaign

**Table 1** Age and sex distribution of melanoma patients and of controls

| Group | Male | | Female | | Total |
|---|---|---|---|---|---|
| | No. | Median (range) | No. | Median (range) | |
| Controls | 19 | 53 (17–70) | 15 | 60 (47–79) | 34 |
| Melanoma stage 1 & 2 | 3 | 28 (18–51) | 0 | – | 3 |
| Melanoma stage 3 | 9 | 51 (35–74) | 6 | 60 (50–74) | 15 |
| Melanoma stage 4 | 5 | 47 (37–57) | 9 | 64 (41–81) | 14 |
| Total | 36 | | 30 | | 66 |

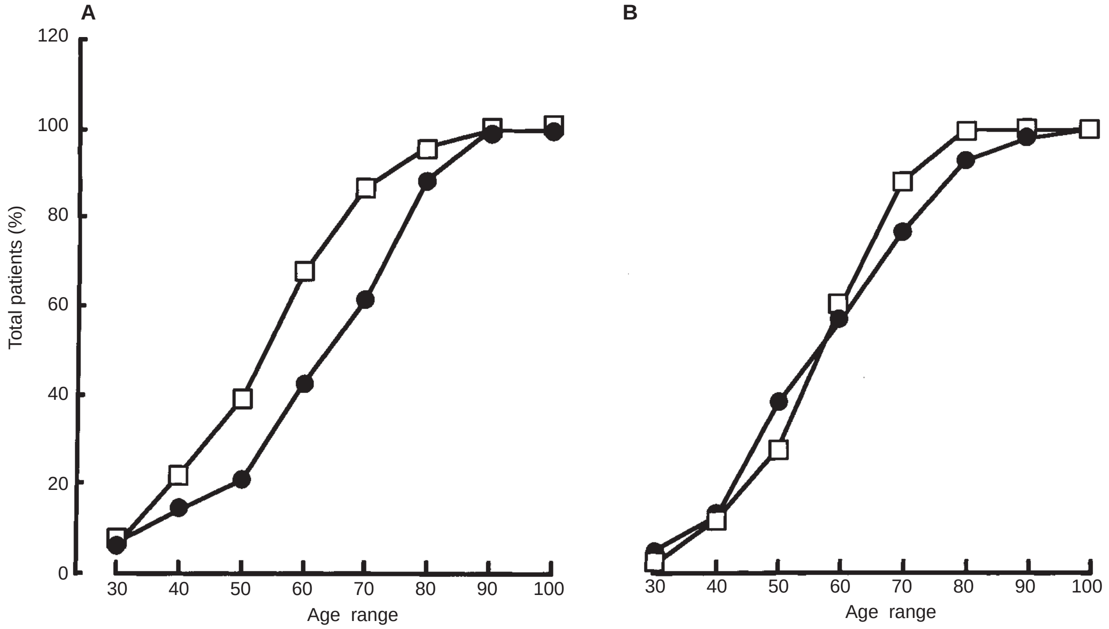

**Figure 1** Comparison of the accumulative age distribution for patients with stage 3 (**A**) and stage 4 (**B**) melanoma attending Weston Park Hospital, Sheffield 1998. Accumulative data for male patients is depicted by (□) and for female patients by (●)

approved by the Ethical Committee of the Northern General Hospital Trust and Weston Park Hospital Sheffield. None of these patients were receiving any treatment at the time of the study. Twenty millilitres of blood was taken, separated into serum and aliquots of serum stored at –80°C until analysis.

Control subjects were age matched and obtained by using excess serum received from subjects who had undergone venesection for routine preoperative assessment screening (five male, four female), cholesterol screening (nine male, six female), pre-cardiac angiography (three male, three female) and non-specific tiredness (two male, two female). Female subjects were also matched for menopausal status using the ratio of FSH/LH. In controls this was 0.35 (95% confidence limits were 0.31–0.4) compared with 0.37 for melanoma patients (95% confidence limits of 0.31–0.43).

### Measurement of sex steroids and sex hormone binding globulin (SHBG)

Leuteinizing hormone (LH), follicle stimulating hormone (FSH), testosterone and oestradiol were measured using chemiluminescent immunoassays from Chiron Diagnostics (Halsted, Essex, UK) on an ACS:180 analyser. The coefficients of imprecision ranged from 5 to 7% for LH, 5–7% for FSH, 6–20% for testosterone and 5–25% for oestradiol. The testosterone assay was selected because it gives good agreement with a reference method (Wheeler et al, 1996; Wians et al, 1997). The oestradiol method was selected on its negligible cross-reactivity with a wide range of natural and synthetic oestrogens. Low concentrations of oestradiol and testosterone were confirmed by repeat analysis.

Oestrone (CV 7–12.5%) was measured using a radioimmunoassay from IDS Ltd. (Bolden, Tyne & Wear, UK). SHBG (CV, 4–8%) was measured by IRMA (Pharmacia & Upjohn, Milton Keynes, UK).

### Statistical analysis

Comparison of levels of steroids, FSH, LH and SHBG between melanoma patients and controls were undertaken by Wilcox rank non-parametric analysis. Students' paired and non-paired *t*-tests were used as appropriate for analysis of in vitro data.

*© 1999 Cancer Research Campaign* 

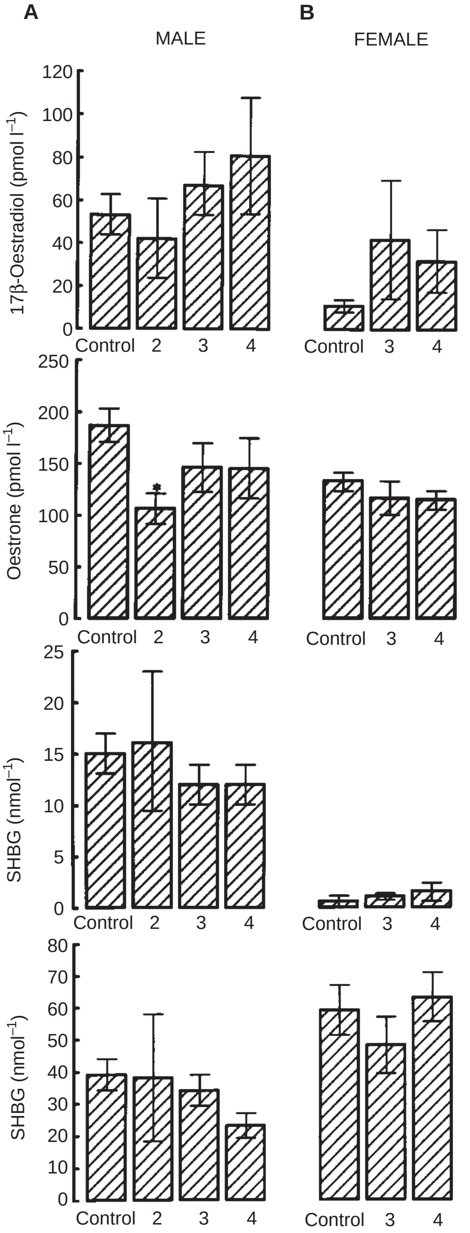

**Figure 2** Comparison of levels of sex steroids and of SHBG in male and female melanoma patients versus disease stage in comparison with aged and sex matched controls. Histograms show means ± s.e.m. of data depicted in Table 2. Values differing significantly from controls are indicated by * = $P < 0.05$ by Wilcoxon rank analysis

### RESULTS

### Retrospective analysis of age and sex distribution for Sheffield melanoma patients

The database of patients with melanoma presenting for treatment in Sheffield Weston Park Hospital was examined initially for the age and sex distribution of patients presenting with melanoma stage 1–4. Of 196 patients on this database, the age distribution for accumulative presentation did not differ for male and female patients presenting with stage 1 and 2 melanoma. However, there were comparatively few patients with stage 1 and 2 melanoma (12 male and eight female attending this clinic). However, for the 69 patients with stage 3 melanoma, 40% of all male patients with stage 3 tumours were aged 50 or less compared to only 20% of all female patients with stage 3 (Figure 1A). For the 56 male and 61 female patients with stage 4 melanoma there was no difference in accumulative age distribution (Figure 1B).

### Prospective study of relationship between sex steroid status and disease progression

Thirty-two volunteers and 34 appropriately age- and sex-matched controls (obtained as described in Materials and Methods) were recruited into a study in which they donated blood for analysis of soluble S-100 and sex steroids and SHBG. For patients, information related to age, menopausal status, medication which might influence sex steroid and receptor levels (e.g. contraceptive pill or hormone replacement therapy) and height and weight (to calculate the body mass index) were also recorded. Table 1 summarizes the age and sex distribution of melanoma patients and controls.

Figure 2 illustrates the data obtained for sex steroids and SHBG versus melanoma stage for male and female patients respectively. From this it can be seen that SHBG levels were lower in men with stage 4 tumours (although this failed to achieve statistical significance, $P < 0.07$) and that oestrone levels were significantly reduced in men with stage 2 tumours ($P < 0.05$) and also lower for all male subjects compared to control male subjects ($P < 0.03$) (not depicted in Figure 2).

Table 2 summarizes the data obtained for male and female patients and controls. For the majority of comparisons, we found melanoma patients differed from age- and sex-matched controls in relatively few respects – as stated oestrone was lower for all male patients compared to male controls and for stage 2 male melanoma patients as a group; SHBG in male stage 4 patients was approximately half the value seen in male controls but failed to achieve statistical significance on Wilcox analysis. The table also shows free androgen index (FAI) (obtained by dividing testosterone by SHBG concentration) and the ratio of oestrogens to androgens (E/A). There were no significant changes in either with disease progression.

### In vitro study of influence of sex steroids on melanoma cell invasion

We initially extended previous studies looking at the influence of tissue culture media on tumour cell invasion. A comparison of six media (as detailed in Table 3) showed that the anti-invasive response to 17β-oestradiol was seen most clearly in RPMI-1640 medium. In the presence of F12:DMEM we failed to see any anti-invasive response to 17β-oestradiol when examined at concentrations up to 1 μM. Responses to 17β-oestradiol in the other media were intermediate between the two. Subsequently, all studies were undertaken using medium RPMI-1640 (phenol-free).

We compared seven structurally related steroids for their ability to influence invasion. Figure 3 illustrates combined data from a minimum of 3–8 experiments with each steroid and Table 4 summarizes the reference ranges for physiological concentrations of sex steroids and summarizes the overall extent of steroid influence on invasion of A375-SM in vitro.

From Figure 3 and Table 4 it is clear that (as previously reported) 17β-oestradiol inhibits invasion in these cells. However,

© 1999 Cancer Research Campaign

**Table 2** Statistical analysis of relationships between sex steroid status and melanoma stage

| Factor | Sex | Melanoma stage | | | |
|---|---|---|---|---|---|
| | | Control | 1 & 2 | 3 | 4 |
| Oestradiol | M | 53 ± 10 | 42 ± 19 | 67 ± 16 | 81 ± 27 |
| (pmol l)$^{-1}$ | F | 11 ± 3 | – | 42 ± 28 | 32 ± 15 |
| Oestrone | M | 186 ± 17 | 106 ± 15[a,b] | 146 ± 24[a] | 145 ± 30[a] |
| (pmol l$^{-1}$) | F | 133 ± 9 | – | 117 ± 16 | 116 ± 8 |
| Total estrogens | M | 239 ± 26 | 148 ± 55 | 214 ± 38 | 227 ± 40 |
| (pmol l$^{-1}$) | F | 145 ± 10 | – | 159 ± 30 | 148 ± 19 |
| Testosterone | M | 15 ± 2 | 16 ± 7 | 12 ± 2 | 12 ± 2 |
| (nmol l$^{-1}$) | F | 0.6 ± 0.5 | – | 1.0 ± 0.4 | 1.6 ± 0.8 |
| SHBG | M | 39 ± 5 | 38 ± 20 | 34 ± 5 | 23 ± 4 |
| (nmol l$^{-1}$) | F | 59 ± 8 | – | 48 ± 9 | 63 ± 8 |
| FAI | M | 46 ± 7 | 48 ± 6 | 42 ± 8 | 53 ± 10 |
| | F | 1.5 ± 0.4 | – | 2.0 ± 0.5 | 3.2 ± 2.5 |
| E/A | M | 19 ± 2.6 | 13 ± 5 | 22 ± 6 | 26 ± 9 |
| | F | 479 ± 117 | – | 602 ± 247 | 275 ± 101 |
| LH | M | 3.9 ± 0.3 | 3.2 ± 1.0 | 4.3 ± 0.8 | 3.0 ± 0.4 |
| (IU l$^{-1}$) | F | 26 ± 3 | – | 41 ± 9 | 28 ± 7 |
| FSH | M | 6 ± 1 | 5 ± 1 | 7 ± 2 | 7 ± 2 |
| (IU l$^{-1}$) | F | 75 ± 7 | – | 110 ± 21 | 73 ± 14 |

[a]Oestrone values for all male patients are significantly lower ($P < 0.05$) than for control values. [b]$P < 0.05$ by Wilcoxon analysis compared to control values.

**Table 3** Effect of media on ability of 17β oestradiol to inhibit invasion of A375-SM melanoma cells

| Media | % Control invasion seen with 1 μM 17β oestradiol | | |
|---|---|---|---|
| | χ ± s.e.m | *n* | Significance |
| F12:DMEM | 100 ± 56 | 3 | NS |
| BME | 61 ± 12 | 3 | $P < 0.05$ |
| F12-HAM | 48 ± 5 | 3 | $P < 0.05$ |
| DMEM | 40 ± 19 | 3 | $P < 0.05$ |
| EMEM | 37 ± 7 | 7 | $P < 0.001$ |
| RPMI-1640 | 27 ± 10 | 5 | $P < 0.001$ |

oestrone was approximately 50 times more potent than 17β-oestradiol in this respect. The other steroids tested, testosterone, androstenedione, progesterone and cortisol did not significantly affect invasion, although results with all of these steroids were slightly above the control level of invasion seen in the absence of steroid. Only in the case of DHEA did the increase in invasion achieve statistical significance as shown in Figure 3 and noted in Table 4.

Confirmation of the anti-invasive effects of oestradiol and oestrone in another cell line was obtained using the HBL human melanoma cell line. Figure 4 illustrates the ability of oestradiol and oestrone to inhibit invasion of this cell line although the potency of both steroids in this cell line was approximately 100-fold less than in the A375-SM cell line.

### In vitro study of influence of sex steroids on melanoma cell proliferation

Figure 5 illustrates the combined data of 6–10 experiments in which A375-SM cells were grown on either tissue culture plastic or fibronectin in the presence of a range of concentrations of dihydrotestosterone, 17β-oestradiol and oestrone. Initial experiments were conducted over 24 and 48 h and in the presence and absence of FCS. Analysis of the data revealed that similar results were obtained irrespective of whether incubation length was 24 or 48 h and whether FCS was present or absent. Accordingly, all results were combined and are shown in Figure 5.

Steroids did not affect proliferation of cells grown on tissue culture plastic (with the exception of higher concentrations of dihydrotestosterone which significantly reduced proliferation of cells) (Figure 5C). Also, fibronectin was not itself mitogenic for these cells (results not shown). However, we found that when cells were grown on fibronectin, there was a modest mitogenic effect of all three steroids. This achieved statistical significance in the case of oestrone, although the stimulation of proliferation was only of the order of 5–10%, did not achieve significance in the case of oestradiol (as variation between experiments was large) but clearly was most marked in response to dihydrotestosterone where the average increase in proliferation was of the order of 40% ($P < 0.05$ at concentrations of $10^{-9}$ to $10^{-6}$ M). (In one particular experiment, dihydrotestosterone induced a 70% increase in proliferation within 24 h.)

### DISCUSSION

The purpose of this study was to obtain more basic information on the extent to which cutaneous melanoma cells might be steroid-sensitive, focusing in particular on whether steroids influence interaction of melanoma cells with extracellular matrix (ECM) proteins.

For the majority of patients with metastatic melanoma, prognosis depends on the depth of extension of malignant melanocytes into the dermis. Clinically, once the melanoma tumour is considered to have begun to metastasize, patients may be offered chemotherapeutic or immunomodulatory therapy to retard metastatic spread or increase the disease-free interval. However, at present, patients presenting with thin tumours are not usually offered any prophylactic treatment following surgical excision. If

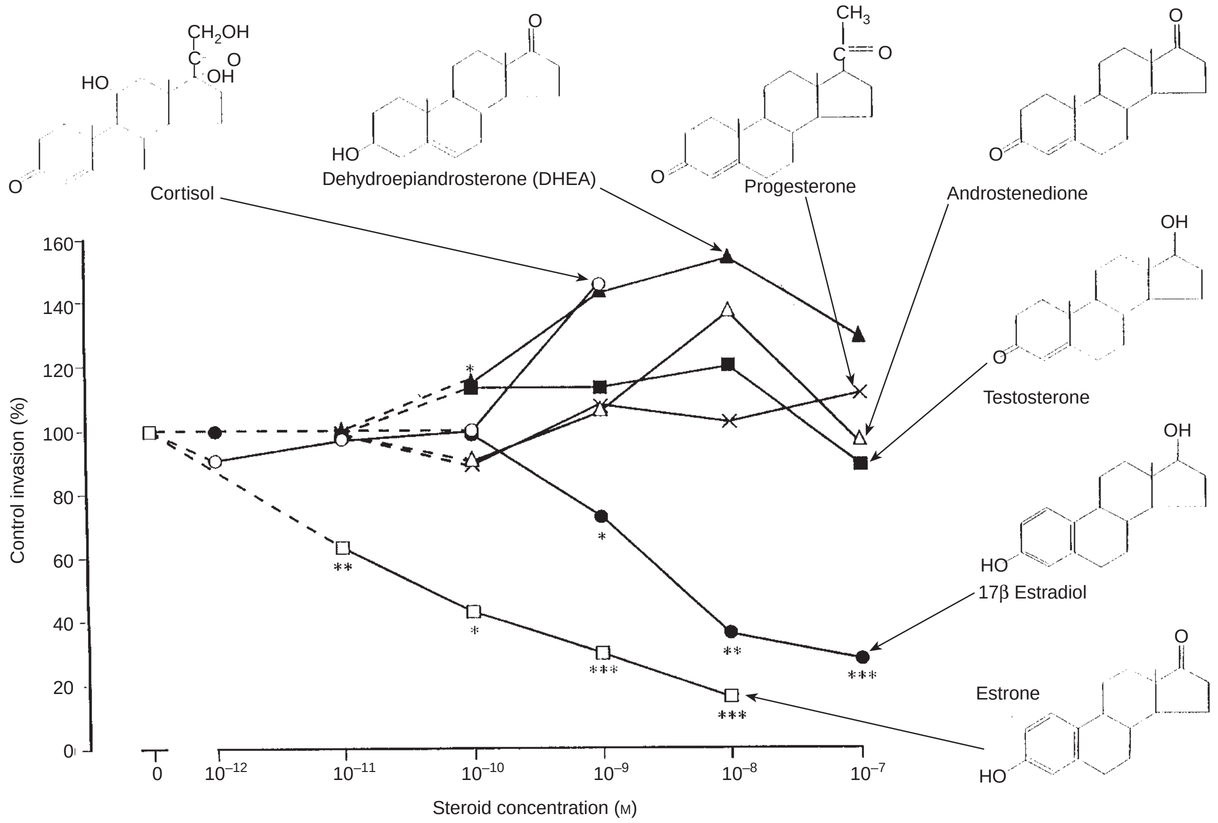

**Figure 3** The effects of steroids on invasion of A375-SM cells through fibronectin. Results show the means ± s.e.m. of 3–8 experiments. Values have been expressed as a percentage of the invasion seen in the absence of any additions. Values differing significantly from invasion in the absence of steroids are indicated by *$P < 0.05$, **$P < 0.01$ and ***$P < 0.001$ as determined by Student's paired *t*-test. The Figure also depicts the structure of these steroids

**Table 4** Physiological ranges of steroids

| Steroid | Physiological concentration range | | | | Overall effect on invasion of A375-SM cells in vitro |
|---|---|---|---|---|---|
| Cortisol | Male | | 138–635 | nM | No significant effect up to 1 nM |
| | Female | a | 138–635 | | |
| | | b | 138–635 | | |
| Progesterone | Male | | 0.4–3.1 | pM | No significant effect up to 100 nM |
| | Female | a | 0.5–79.5 | | |
| | | b | 0.5–2.2 | | |
| DHEA | Male | | 0.3–16.7* | nM | Significant stimulation at 1 nM (143 ± 12, $P < 0.05$) |
| | Female | a | 0.8–21.1 | | |
| | | b | 0.8–7.0 | | |
| Androstenedione | Male | | 2.6–7.2 | nM | No significant effect up to 100 nM |
| | Female | a | 3.0–9.6 | | |
| | | b | 3.0–9.6 | | |
| Testosterone | Male | | 0.5–38.2 | nM | No significant effect up to 100 nM |
| | Female | a | 0.5–2.4 | | |
| | | b | 0.5–2.4 | | |
| 17β oestradiol | | Male | 37–184 | pM | Significant inhibition from 1 nM |
| | Female | a | 73–2753 | | |
| | | b | <73 | | |
| Oestrone | Male | | 55–240 | pM | Significant inhibition from 10 pM |
| | Female | a | 55–925 | | |
| | | b | 55–204 | | |

a, Range for pre-menopausal women. b, Range for post-menopausal women. * Male levels also fall with age, e.g. > 50 (0.3–11.1)

 *© 1999 Cancer Research Campaign*

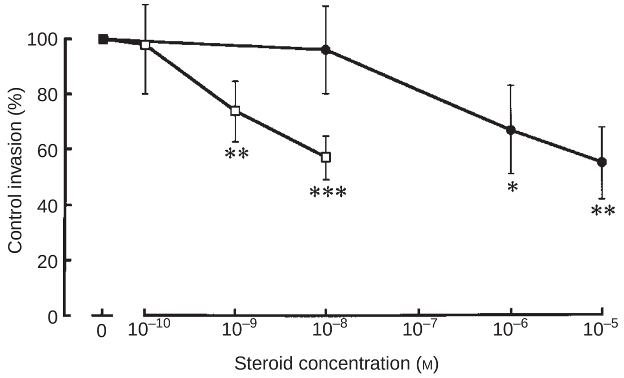

**Figure 4** Comparison of the ability of 17β-oestradiol (●) and oestrone (□) to inhibit invasion of the HBL melanoma cell line. Results show the mean ± s.e.m. of 3–4 experiments. Values have been expressed as a percentage of the invasion seen in the absence of any additions. Values differing significantly from invasion in the absence of drugs are indicated by $*P < 0.05$ and $**P < 0.01$ and $***P < 0.001$ as determined by Student's paired *t*-test

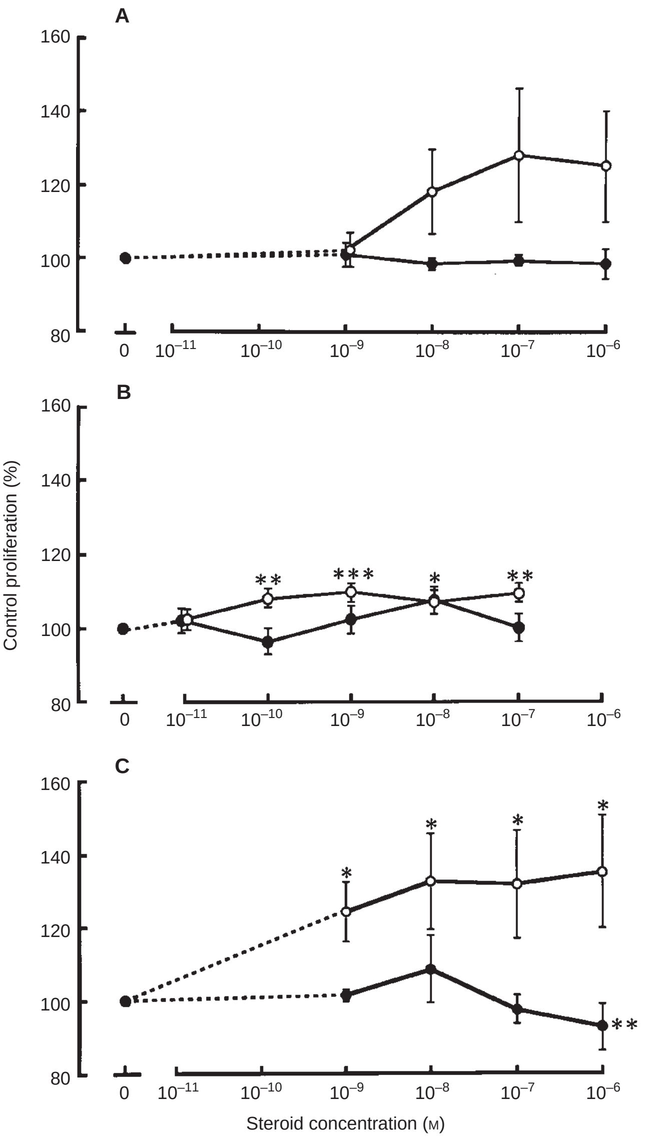

**Figure 5** Effect of steroids on proliferation of A375-SM cells cultured on tissue culture plastic (●) or on fibronectin (○). Results shown are for (**A**) oestradiol, (**B**) oestrone and (**C**) dihydrotestosterone. Values shown are means ± s.e.m. expressed as a percentage of the proliferation in the absence of steroid. Results shown are means of 6–10 experiments. Values differing significantly from proliferation in the absence of steroid are indicated by $*P < 0.05$, $**P < 0.01$ and $***P < 0.001$ by Student's paired *t*-test sex steroids can affect the likelihood of tumours metastasizing then this is worth investigating further because of the potential it offers with respect to prevention of initial metastasis.

For these reasons we have looked in some detail at a number of aspects of patients circulating sex steroid status and tumour progression and the direct effects of sex steroids on melanoma proliferation and invasion in vitro.

The patients attending Weston Park Hospital for treatment for metastatic melanoma did not differ with respect to age and sex when presenting with thin early stage tumours (stage 1 and 2). However, there were relatively few patients with early stage melanoma attending this clinic. There was a noticeable age difference in the male and female patients for stage 3 melanoma. Using age 50 as an approximate indicator of menopause, twice as many men had presented with stage 3 by age 50 compared to women by age 50. However, when one looked at the age distribution of patients with stage 4 melanoma, no sex difference was seen.

We suggest that one interpretation of this data is that any female advantage in melanoma cannot be seen if one looks at patients presenting with early stage (thin melanoma or adjacent metastasis). However, there is an interesting discrepancy in the stage 3 melanoma sex distribution. Melanomas may progress to stage 3 more slowly in female than male patients. Subsequent progression from stage 3 to stage 4 melanoma does not show any evidence of a female survival advantage. We recognize this is a relatively crude analysis of this database, but suggest that it does reflect the clinical epidemiology seen in many other larger databases which show progression in premenopausal females is slower than in males or post-menopausal women (Cocconi et al, 1992). Thus, oestrogens could be responsible, at least in part, for the delayed progression to stage 3 disease in the women. However, this suggestion requires analysis using a much larger database of patients with ideally a progressive study of menopausal status and disease progression. We recognise that many factors influence melanoma progression and that the disease does not necessarily progress through each stage in every patient also.

For the cohort of patients for whom we investigated their circulating sex steroid and sex hormone binding globulin status, we found relatively few differences overall. Two differences that we did find and were unable to explain in terms of patient age or body mass index were a decrease in SHBG in male patients with advanced melanoma (which just failed to achieve statistical significance, $P < 0.07$) and a significant decrease in circulating levels of oestrone in male patients. The reduced SHBG in male patients with melanoma confirmed this finding in our previous study (Miller et al, 1997). The reduced levels of SHBG in male patients with stage 4 disease did not relate to any decrease in body mass index which we excluded for these patients (results not shown).

There is currently no known role of SHBG in melanoma. Perhaps the most important property of SHBG is that it regulates the levels of free androgens and oestrogens in the serum, thereby controlling diffusion of the free fraction of steroids from plasma into the tissues. It binds 5α–dihydrotestosterone and testosterone with high affinity and oestradiol with lower affinity (Petra, 1991).

The production of SHBG by the liver is influenced by several factors. Androgens decrease the production of SHBG by the liver, but oestrogens, thyroid hormones and insulin all increase the production of SHBG. Increasing amounts of oestrogen can have a devastating effect on the bio-availability of androgens. As oestrogen concentration increases so does the SHBG level. Increasing amounts of SHBG bind testosterone thus decreasing bio-available testosterone. In vitro studies have shown that the addition of pure SHBG to endometrial cancer cells maintained in culture appear to inhibit the incorporation of 17β-oestradiol into cancer cells (De Ryck et al, 1985). This supports the premis that the unbound steroid fraction enters into cells and represents the active form of the hormone. A decrease in SHBG in stage 4 melanoma for male patients might suggest that melanoma cells would be exposed to greater levels of male sex hormones in this late-stage disease. Interestingly, large increases in SHBG have been reported as a long-term effect of tamoxifen therapy for breast cancer (Jordan et al, 1987; Cuzick et al, 1993). Also a direct role of SHBG in transporting sex steroids across the plasma membrane cannot be ruled out. Rosner et al (1991) have demonstrated that SHBG has its own receptor sites on plasma membranes. If the SHBG binding site is occupied by a steroid then it cannot bind to its receptor. However, if unliganded SHBG is allowed to bind to its receptor on intact cells and an appropriate biologically active steroid hormone is then introduced the second messenger cyclic AMP is increased. Thus Rosner et al have demonstrated an additional mode of action of steroid hormone, one that does not require that the steroid interacts with the steroid receptor.

Differences between the sexes in the patient and volunteer groups that were unrelated to disease progression were obviously due to the very high free androgen index in all male volunteers in the study compared to female volunteers. The oestrogen/androgen index was decreased in stage 4 females, but the variation was such that this did not achieve statistical significance. However, the current study leaves open the possibility that oestrogens may retard melanoma progression and that conversely androgens may actively stimulate aspects of tumour growth. (We also, in this study, measured soluble S-100 to use this hopefully as an indicator of disease stage. However, we were unable to confirm its usefulness in this respect in this study, as all but four controls and four patients showed levels of S-100 beneath the limits of detection. On investigation, control subjects were not found to be suffering from conditions likely to elevate S-100, results not shown.)

Turning next to the question of whether melanoma tumours themselves contain oestrogen receptors, we have previously failed to find convincing evidence of oestrogen receptor-α in a large number of melanoma tumours or in the A375-SM cell line used in this study (Dewhurst et al, 1997; Miller et al, 1997). However, the recent cloning of oestrogen receptor-β offers an explanation to the conflicting data in the literature concerning binding of radiolabelled oestradiol in the absence of any specific oestrogen receptor-α receptors (Duncan et al, 1994; and as discussed in Miller and Mac Neil, 1997).

At present, the function of ERβ is under intense investigation. Early reports suggest that acting at the level of transcription factors, it affects gene transcription with quite a different profile of gene regulation to oestrogen receptor-α. Skin has previously been reported to be positive for ERβ although at low levels (Brandenberger et al, 1997). It does not appear that melanoma has yet been investigated for the ERβ mRNA or the ERβ protein. In view of the absence of oestrogen receptor-α in the A375-SM cell line and its responsiveness to oestrogens (as shown in this study in terms of both invasion and proliferation) and the epidemiological evidence showing female survival benefit in melanoma despite the lack of oestrogen receptor-α in the majority of tumours, this is obviously an area which merits investigation.

In looking at the effects of steroids on invasion of cutaneous melanoma cells in vitro, we have, as in our first report on this area (Dewhurst et al, 1997), used a relatively simple model of melanoma cells invading through fibronectin. Fibronectin is normally absent or at very low concentrations in normal skin but will be produced rapidly by dermal fibroblasts as part of a transitional matrix in wound healing. Melanoma cells have been found to secrete soluble factors which are capable of influencing the glycosaminoglycan component of the extracellular matrix produced by fibroblasts (Edwards et al, 1992). There are also a number of immunohistochemical reports consistent with melanoma cells inducing considerable turnover and breakdown of matrix associated with invasion.

We undertook experiments looking at the possible mitogenic effect of steroids on A375-SM cells both on tissue culture plastic and on fibronectin primarily to examine whether any mitogenic effects of the steroids were likely to occur in the invasion assay (albeit this is only 20-h duration). We were surprised to discover that the steroids we examined did not affect melanoma proliferation when cells were grown on tissue culture plastic but did when cells were grown on fibronectin. Fibronectin itself was not mitogenic for these cells hence our data gives clear evidence of a synergism of the effects of fibronectin and of steroids (dihydrotestosterone, in particular) in inducing proliferation of melanoma cells. Steroids influence wound healing, e.g. wound healing in pregnancy is often exaggerated and hypertrophic scars and hyperpigmentation are relatively common. While the extent of the mitogenic response to the steroids is modest under the circumstances so far investigated in vitro, we now need to conduct further work to explore longer periods of exposure to the steroids in the presence of a range of matrix proteins.

When we looked at the effect of seven different steroids on A375-SM melanoma cell invasion, the results were very clear as only the two oestrogenic compounds significantly reduced invasion. It was also obvious that there was a slight (although extremely variable) enhancement of invasion with some of the androgenic compounds. This only achieved statistical significance for DHEA due to the considerable variation seen in these assays. The finding that DHEA positively affected invasion is interesting from the structural viewpoint. Unlike the other androgens tested, it has a double bond between the 5 and 6 carbon atoms as opposed to the 4 and 5 carbon atoms in the other androgens. We, therefore, have a situation where the structure of the A ring of the steroids appears to influence the invasion of melanoma cells. An A ring fully aromatized as in the oestrogens seems to inhibit invasion. A complete lack of double bonds in the A ring as in DHEA seems to enhance invasion. Although we would suggest that these results based on one simple in vitro assay be interpreted with caution at this stage, these data combined with the mitogenic effect of dihydrotestosterone with fibronectin on melanoma cells lead us to suggest that this work be extended into other models of invasion both in vitro and in vivo. Certainly, the current work is consistent with oestrogenic compounds being anti-invasive and androgenic compounds clearly not sharing this property. Progesterone did not reduce invasion of melanoma cells in these experiments.

 © 1999 Cancer Research Campaign

While this study has been extremely useful in identifying which steroids can affect melanoma cell invasion, the levels of these steroids and SHBG now need to be studied in combinations which reflect the circulating steroid status of the premenopausal versus the post-menopausal woman, the pregnant female and the young versus the old male.

We have not, in this study, looked into the mechanism of how oestradiol or oestrone affects melanoma cell invasion through fibronectin and this also remains to be investigated. However, irrespective of the details of the mechanism, the implications of the current study are that further work in this area might lead to the development of a prophylactic approach to preventing melanoma cells interacting effectively with ECM proteins and invading from the site of the initial tumour.

### ACKNOWLEDGEMENTS

We thank the Yorkshire Cancer Research Campaign for funding this study. We gratefully acknowledge the contribution of LH, FSH, testosterone and oestradiol chemiluminescent assays from Chiron Diagnostics for this study.

### REFERENCES

Brandenberger AW, Tee MK, Lee JY, Caho V and Jaffe RB (1997) Tissue distribution of oestrogen receptors alpha and beta mRNA in the midgestational human foetus. *J Endocrinol Metab* **82**: 3509–3512

Breslow A (1970) Thickness, cross-sectional areas and depth of invasion in the prognosis of cutaneous melanoma. *Ann Surg* **172**: 902–908

Cocconi G, Bella M, Calabresi F, Tonato M, Canaletti, Boni C, Buzzi F, Ceci G, Corgna E, Costa P, Lottici R, Papadia F, Sorfa MC and Bacchi M (1992) Treatment of metastatic malignant melanoma with dacarbazine plus tamoxifen. *N Engl J Med* **327**: 516–523

Cuzick J, Allen M, Baum J, Barrett J, Clark G, Kakkar V, Melissari E, Moniz C, Moore J, Parsons V, Pemberton K, Pitt P, Richmond W, Houghton J and Riley D (1993) Long-term effects of tamoxifen. *Eur J Cancer* **29A**: 15–21

Daffada AAI, Johnston SRD, Smith IE, Detre S, King N and Dowsett M (1995) Exon 5 deletion variant estrogen receptor messenger mRNA expression in relation to tamoxifen resistance and progesterone receptor/pS2 status in human breast cancer. *Anticancer Res* **55**: 288–293.

De Ryck L, Ross JBA, Petra PH and Gurpide E (1985) Oestradiol entry into endometrial cells in suspension. *J Steroid Biochem* **23**: 145–152

Dewhurst LO, Gee JW, Rennie IG and Mac Neil S (1997) Tamoxifen, 17β oestradiol and the calmodulin antagonist J8 inhibit human melanoma cell invasion through fibronectin. *Br J Cancer* **75**: 860–868

Duncan LM, Travers RL, Koerner FC, Mihm MC and Sober AJ (1994) Estrogen and progesterone receptor analysis in pregnancy associated melanoma: absence of immunohistochemically detectable hormone receptors. *Hum Pathol* **25**: 36–41

Edwards M, Grant AW and MacKie RM (1992) Human melanoma cell-derived factor(s) stimulates fibroblast glycosaminoglycan synthesis. *Int J Cancer* **52**: 499–503

Feucht KA, Walker MJ, Das Gupta TK and Beattie CW (1988) Effect of 17β oestradiol on the growth of estrogen receptor positive human melanoma in vitro and in athymic mice. *Cancer Res* **48**: 7093–7101

Gefeller O, Hassan K and Wille L (1998) Cutaneous malignant melanoma in women and the role of oral contraceptives. *Br J Dermatol* **138**: 122–124

Jordon VC, Fritz NF and Tormey DC (1987) Long-term adjuvant therapy with tamoxifen: effects on sex hormone binding globulin and antithrombin III. *Cancer Res* **47**: 4517–4519

Karjalainen S and Hakulinen T (1988) Survival and prognostic factors of patients with skin melanoma: a regression model analysis based on Nationwide Cancer Registry data. *Cancer* **62**: 2274–2280

Ladanyi A, Timar J, Bocsi J, Tovari J and Lapis K (1995) Sex-dependent liver metastasis of human melanoma lines in SCID mice. *Melanoma Res* **5**: 83–86

Luqmani YA, Smith J and Coombes RC (1992) Polymerase chain reaction-aided analysis of gene expression in frozen tissue sections. *Ann Biochem* **200**: 291–295

MacKie RM, Bufalino R, Morabito A, Sutherland C and Cascinelli N (1991) Lack of effect of pregnancy on outcome of melanoma. *Lancet* **337**: 633–635

Mac Neil S, Wagner M, Kirkham PR, Blanksom EA, Lennard MS, Goodall T and Rennie IG (1993) Inhibition of melanoma cell/matrix interaction by tamoxifen. *Melanoma Res* **3**: 67–74

Miller JG, Gee J, Price A, Wagner M and Mac Neil S (1997) Investigation of oestrogen receptors, sex steroids and soluble adhesion molecules in the progression of malignant melanoma. *Melanoma Res* **7**: 197–208

Miller JG, Mac Neil S (1997) Gender and cutaneous melanoma. *Br J Dermatol* **136**: 657–665

Morvillo V, Luthy IA, Bravo AI, Capurro MI, Donaldson M, Quintans C, Calandra RS and Mordah J (1995) Atypical androgen receptor in the human cutaneous melanoma cell line 11B-Mel-J. *Pigment Cell Res* **8**: 135–141

O'Brian JP, Frye RA, Cogswell PC, Neubauer A, Kitch B, Prokop C, Espinosa R, Le Beau MM, Earp HS and Liu ET (1991) Axl, a transforming gene isolated from primary human myloid leukemia cells, encodes a novel receptor tyrosine kinase. *Mol Cell Biol* **11**: 5016–5031

Petra PH (1991) The plasma sex steroid binding protein. A critical review of recent developments on the structure, molecular biology and function. *J Steroid Biochem Mol Biol* **40**: 75–753

Rosner W, Hryb D, Khan S, Nakhla AM and Romas N (1991) Sex hormone binding globulin: anatomy and physiology of a new regulatory system. *J Steroid Biochem Mol Biol* **40**: 813–820

Shaw HM, McGovern J, Milton GW, Farago G and McCarthy WH (1980) Malignant melanoma: influence of site of lesion and age of patient in the female superiority in survival. *Cancer* **46**: 2731–2735

Shaw HM, Milton GW, Farago G and McCarthy WH (1978) Endocrine influences on survival from malignant melanoma. *Cancer* **42**: 669–677

Slingluff CL and Seigler HF (1992) Malignant melanoma and pregnancy. *Ann Plast Surg* **28**: 95–99

Stidham KR, Johnson JL and Seigler HF (1994) Survival superiority of females with melanoma: a multivariate analysis of 6383 patients exploring the significance of gender in prognostic outcome. *Arch Surg* **129**: 316–324

Travers RL, Sober AJ, Berwick M, Mihm Jr MC, Barnhill RL and Duncan LM (1995) Increased thickness of pregnancy-associated melanoma. *Br J Dermatol* **132**: 876–883

Vladusic EA, Hornby AE, Guerra-Vladusic FK and Lupu R (1998) Expression of estrogen receptor ss messenger in breast cancer. *Cancer Res* **58**: 210–214

Wheeler MJ, De Souza A, Matadeen J and Croos P (1996) Ciba Corning ACS:180 testosterone assay evaluated. *Clin Chem* **42**: 1445–1449

Wians FH and Stuart J (1997) Ciba Corning ACS:180 Direct total testosterone assay can be used on female sera. *Clin Chem* **43**: 1466–1467

© 1999 Cancer Research Campaign 
